# Assessment of a fragment of e-cadherin as a serum biomarker with predictive value for prostate cancer

**DOI:** 10.1038/sj.bjc.6602599

**Published:** 2005-05-03

**Authors:** R Kuefer, M D Hofer, C S M Zorn, O Engel, B G Volkmer, M A Juarez-Brito, M Eggel, J E Gschwend, M A Rubin, M L Day

**Affiliations:** 1 Department of Urology, Faculty of Medicine, University of Ulm, Prittwitzstrasse 43, Ulm 89075, Germany; 2 Department of Urology and the O'Brien Cancer Center, University of Michigan, Ann Arbor, MI 48109, USA; 3 Department of Pathology, Brigham and Women's Hospital, Harvard University, School of Medicine, Boston, MA 2500, USA

**Keywords:** e-cadherin, human prostate cancer, serum biomarker, 80 kDa fragment, progression

## Abstract

In prostate cancer, biomarkers may provide additional value above standard clinical and pathology parameters to predict outcome after specific therapy. The purpose of this study is to evaluate an 80 kDa fragment of the cell adhesion molecule e-cadherin as a serum biomarker. A broad spectrum of prostate cancer serum samples, representing different stages of prostate cancer disease, including benign prostatic hyperplasia (BPH), localised (Loc PCA) and metastatic prostate cancer (Met PCA), was examined for the cleaved product. There is a significant difference in the expression level of the 80 kDa fragment in the serum of healthy individuals *vs* patients with BPH and between BPH *vs* Loc PCA and Met PCA (*P*<0.001). Highest expression levels are observed in advanced metastatic disease. In the cohort of Loc PCA cases, there was no association between the 80 kDa serum concentration and clinical parameters. Interestingly, patients with an 80 kDa level of >7.9 *μ*g l^−1^ at the time of diagnosis have a 55-fold higher risk of biochemical failure after surgery compared to those with lower levels. This is the first report of the application of an 80 kDa fragment of e-cadherin as a serum biomarker in a broad spectrum of prostate cancer cases. At an optimised cutoff, high expression at the time of diagnosis is associated with a significantly increased risk of biochemical failure, potentially supporting its use for a tailored follow-up protocol for those patients.

Prostate-specific antigen (PSA) screening has revolutionised early detection of prostate cancer. Still, prostate cancer is a leading cause of male cancer-related deaths in the western world. Men with clinically localised prostate cancer have excellent chances for long-term cure. However, treatment options are rare in advanced disease stage or in case of failure after specific therapy. Therefore, it is crucial to identify the potentially progressing prostate cancer at its earliest stage. One way to evaluate patient risk is to use standard nomograms, which take clinical and pathology parameters into account. Nomograms have been developed, for example, for predicting the 5-year probability of treatment failure among men with clinically localised prostate cancer treated with radical prostatectomy (Kattan *et al*, 1998), and the probability of successful treatment 5 years after brachytherapy for clinically localised prostate cancer (Kattan *et al*, 2001). Ideally, nomograms would help clinicians to identify progressing tumours after specific therapy, indicating early adjuvant therapeutic means or at least supporting a more tailored follow-up regimen. There appears to be significant opportunity to identify biomarkers to strengthen currently available nomograms by providing information with respect to tumour biology at the time of diagnosis (Potter *et al*, 2001).

The 120 kDa full-length e-cadherin is known to be important for well-functioning cell–cell adhesion and its cleavage has been linked to the malignant progression of adenocarcinomas including prostate cancer (Giroldi and Schalken, 1993; Rios-Doria *et al*, 2002). In a recent study, an 80 kDa fragment of full-length e-cadherin has been described as almost exclusively being observed in the neoplastic aspect of prostate cancer tissue (Kuefer *et al*, 2003). This soluble fragment has also been reported as being measurable in the serum of patients suffering from different types of adenocarcinoma (Katayama *et al*, 1994a; Banks *et al*, 1995; Gofuku *et al*, 1998; Velikova *et al*, 1998; Protheroe *et al*, 1999; Chan *et al*, 2003). In the current study, we report the accumulation of this 80 kDa fragment of e-cadherin in the serum of patients affected by prostate cancer. Its potential use as a serum marker with predictive value for prostate cancer progression is elucidated.

## MATERIALS AND METHODS

### Sample collection

Serum samples were taken from the radical prostatectomy series and from the Rapid Autopsy Program at the University of Michigan. The serum was collected as a standard procedure of all individuals undergoing surgery. In order to study hormone refractory prostate cancer, a Rapid Autopsy Protocol was developed. As a routine procedure, blood is taken from these patients with far advanced disease on a regular basis till the time of autopsy. Of all patients, blood samples were taken and immediately processed and stored at −80°C. Clinical and pathology data for all patients was acquired with approval from the Institutional Review Board (IRB) at the University of Michigan and maintained on a secure relational database.

In this retrospective setting, serum samples were grouped according to following clinical categories in order to examine the widest range of prostate cancer disease: one group consisted of 13 healthy young volunteers. The second group was represented by 29 patients with benign prostatic hyperplasia (BPH). The third group consisted of 109 patients with localised prostate cancer (Loc PCA). Of these patients, serum at the time of diagnosis was available and none of these patients failed during follow-up after radical prostatectomy. The fourth group included 21 cases with localised PCA and biochemical failure after surgery during follow-up. The last cohort consisted of 16 serum samples of different patients with generalised metastatic disease (Met PCA). Detailed clinical data of all patients are given in Table 1.

In this study, Loc PCA is defined as all tumour stages according to the current TNM classification (T1–T4). Patients with node-positive disease were not included due to the small number of cases. Also, patients with other malignancies besides PCA or chronic inflammatory diseases (Katayama *et al*, 1994a) were excluded. None of the patients had neoadjuvant therapy and none of the patients with localised PCA received other cancer-specific treatment besides radical prostatectomy.

### Serum analysis of the 80 kDa e-cadherin fragment

Serum concentrations of the 80 kDa fragment were analysed using the Human E-cadherin EIA Kit from Zymed Laboratories, San Francisco, USA. In this commercially available kit, HECD-1 is the e-cadherin-specific antibody. HECD-1 is highly specific and known to map to the extracellular domain of e-cadherin, where the 80 kDa fragment derives from (Kuefer *et al*, 2003). In the serum, the soluble 80 kDa is the only cleaved product being seen by the HECD-1 antibody. As first step, a standard curve was created using the suppliers lyophilised human e-cadherin. To this standard curve, a curve was fitted to convert the detected optical densities (OD) into 80 kDa concentrations of the individual serum samples. In this setting, a cubic model with the formula (1.1305 × OD)+(−0.6712 × OD^2^) +(0.1624 × OD^3^) was the optimal model to fit the standards (RSQ=0.981).

Measurements of samples with an OD ⩾3.0 were repeated using higher dilutions. The kit was tested for low intrasample and interplate variability. A representative set of samples was also tested in a time-course experiment to rule out an association between time till the samples were processed and the amount of the cleaved e-cadherin fragment.

Absorbance was measured at 450 nm using a standard 96-well microtiter plate reader. Measurement was carried out in triplicate for each sample and the mean value was used for statistical analysis.

### Statistical analyses

The OD values were imported into SPSS (SPSS, Chicago, USA) and transformed into 80 kDa serum concentrations according to the above formula for statistical analysis. The expression levels of the 80 kDa serum fragment were evaluated for all serum samples (i.e. healthy volunteers, BPH, Loc PCA, Met PCA) and are graphically presented as error bars with 95% confidence intervals (CI). A comparison of expression levels between different cohorts (i.e. BPH *vs* Loc PCA) was performed using the Mann–Whitney test, or the Wilcoxon's signed-rank test for dependent samples. For the Loc PCA samples, the 80 kDa serum expression was investigated for association with clinical and pathological parameters (e.g. tumour stage) using Cramer's V for categorised values. Cramer's V represents a correlation coefficient; the lower (towards 0) the less correlation is given and the higher the value (towards 1), the more correlation is present. Kaplan–Meier analysis was performed to estimate survival. As surrogate end point for survival, biochemical recurrence during follow-up was chosen, being defined as two consecutive PSA rises after a nadir of undetectable PSA after surgery. Univariate, unmatched comparisons of survival were carried out using the log-rank test. The Wald test was used for Cox's hazards regression analysis applying a backward stepwise elimination of variables in multivariate models. Risk ratios (relative hazards) were calculated based on the Cox model and are presented with 95% CI. Values of *P*<0.05 were considered significant.

## RESULTS

### Expression of the 80 kDa fragment for different clinical categories

**Figure 1:**
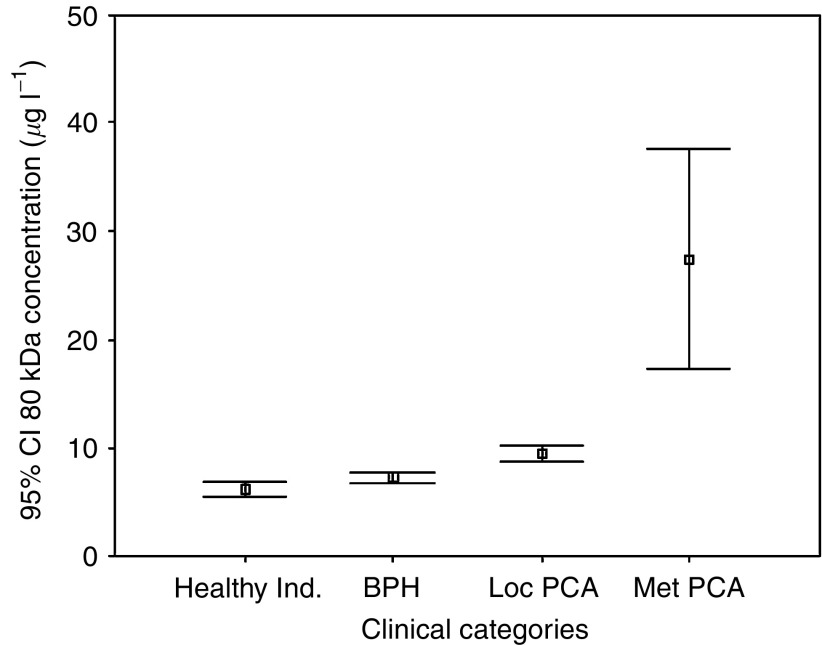
Detection of the 80 kDa fragment of e-cadherin in the serum of Healthy Individuals (Healthy Ind.), patients with benign prostatic hyperplasia (BPH), patients with localised prostate cancer (loc PCA) and with metastatic disease (Met PCA). Mean concentrations including the 95% confidence interval (CI) are given.

The serum levels of healthy volunteers (*n*=13) was compared to patients with BPH (*n*=29) and patients with Loc PCA (*n*=130) and those with Met PCA (*n*=16). Healthy volunteers, BPH, Loc PCA and Met PCA had a mean 80 kDa concentration of 6.27 (standard error (s.e.) 0.31, 95% CI 5.59–6.96), 7.26 (s.e. 0.21, CI 6.82–7.70), 9.46 (s.e. 0.42, CI 8.63–10.29) and 27.49 (s.e. 4.81, CI 17.23–37.75), respectively. Pairwise comparison revealed a statistically significant difference between the serum concentration of the healthy volunteers and the patients with BPH (Mann–Whitney; *P*=0.023) and between patients with BPH and Loc PCA (Mann–Whitney; *P*=0.011). Interestingly, there was a highly significant difference between the Loc PCA cases and the serum levels of the patients with metastatic disease (*P*<0.001). The mean 80 kDa protein expression level for each category is presented in Figure 1, demonstrating a cascade from healthy volunteers <BPH<Loc PCA<Met PCA.

### Expression of the 80 kDa fragment for patients with Loc PCA

#### Association of the 80 kDa expression with clinical and pathological parameters

As the 80 kDa fragment has been found to be associated with aggressive clinical course in bladder, gastric and colorectal cancer (Griffiths *et al*, 1996; Gofuku *et al*, 1998; Velikova *et al*, 1998), we looked for potential associations between clinical or pathological parameters in prostate cancer. Two statistical approaches were tested: first step was to create four categories of the 80 kDa serum concentration according to the calculated quartiles; second step was to categorise using the median of the 80 kDa concentration of the cohort of Loc PCA patients. These categories of the serum biomarker were compared to the categorised clinical and pathological parameters. There was no association between expression of the e-cadherin fragment in the serum and any of the following parameters: age, race, preoperative PSA, tumour stage, Gleason score (categorised Gleason sum <7; 7; >7), multifocality, seminal vesicle involvement, surgical margin, extraprostatic extension and weight of prostate gland (Cramer's V; values ranged from 0.18 to 0.29)

#### Comparison of the 80 kDa serum level at the time of diagnosis and at the time of PSA nadir after radical prostatectomy

For this analysis, serum at the time of diagnosis and at the time of nadir after surgery was available of 21 patients (matched pairs). The mean 80 kDa concentration at the time of diagnosis was 8.27 (s.e. 0.68, 95% CI 6.38–9.54) and 7.32 (s.e. 0.88, CI 5.22–9.13) during follow-up. The difference was statistically not significant (Wilcoxon test; *P*=0.28).

#### Comparison of the 80 kDa serum expression at the time of diagnosis and at the time of failure during follow-up

For this analysis, serum at the time of diagnosis and at the time of failure was available of seven patients (matched pairs) out of 21 patients who failed. The mean 80 kDa concentration at the time of diagnosis was 8.33 (s.e. 0.69, 95% CI 6.65–10.01) and 7.57 (s.e. 0.87, CI 5.44–9.73) at the time of failure. Statistically there was no significant difference in expression levels (Wilcoxon test; *P*=0.31).

#### Outcome analysis for the 80 kDa serum expression level in the Loc PCA group (*n*=130)

**Figure 2:**
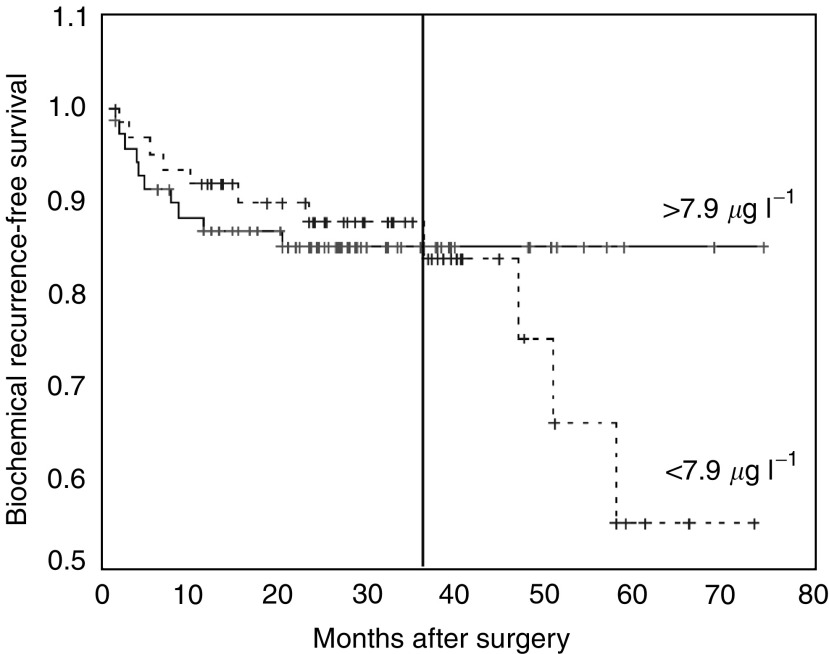
Association of the soluble 80 kDa fragment (cutoff expression level 7.9 *μ*g l^−1^) at the time of diagnosis of prostate cancer and biochemical failure after radical prostatectomy. Patients with localised disease and with high expression levels prior to surgery have a significantly increased risk of late biochemical failure (after 3 years) during follow-up.

The median postsurgery follow-up time was 920.5 days (41–2706 days). The average time to failure was 435.3 days (s.e. 115.5 days). Clinical parameters and the 80 kDa fragment as a serum biomarker were evaluated for their ability to predict outcome. For statistical analysis, a cutoff for the 80 kDa serum level was set at the calculated median of 7.93 *μ*g l^−1^ (mean 9.46 *μ*g l^−1^; s.e. 0.42; CI 8.63–10.29). Tumour stage, Gleason sum (categorised <7; 7; >7), extraprostatic extension, seminal vesicle involvement and surgical margin showed a strong association with outcome (log rank; *P*<0.002). Preoperative PSA, age, multifocality of the prostate cancer and race did not show an association with outcome (log rank; *P*>0.2). In the multivariate Cox regression analysis, applying a backward model selection for all the given parameters, Gleason sum and extraprostatic extension of the cancer had the best model performance (log rank; *P*=0.007). Detailed data of the Cox proportional-hazard model for all the clinical parameters including confidence intervals is given in Table 2. As in the case of the 80 kDa e-cadherin fragment, at first glance there was no association between the serum level and PSA failure (log rank; *P*=0.79, 95% CI 0.48–2.65). Interestingly, when performing a subanalysis, starting at a follow-up time of 35 months, the 80 kDa level was associated with outcome (log rank; *P*<0.05). In this model, the relative risk for PSA failure was 55.1 (s.e. 4.14). The association between the 80 kDa expression and clinical failure is presented graphically in a Kaplan–Meier curve demonstrating a significantly higher risk of PSA failure for patients with 80 kDa serum levels >7.9 *μ*g l^−1^ once a follow-up time of 3 years has passed (Figure 2).

## DISCUSSION

Without doubt, PSA is one of the best tumour markers in oncology. It was first identified by Hara *et al* in 1971 in seminal plasma and some years later in prostate tissue (Wang *et al*, 1979). In the last 20 years screening, treatment approaches and follow-up for prostate cancer have been dramatically influenced by the widespread usage of PSA. Owing to the high prevalence of prostate cancer, tumour marker research still is particularly challenging as PSA is known to have some limitations in its application especially in the diagnostic window of 4–10 ng ml^−1^ (Partin *et al*, 1990). In fact, in combination with other clinical parameters PSA helps to predict locally advanced or metastatic disease (Sands *et al*, 1994; Partin *et al*, 1997; Cagiannos *et al*, 2003), whereas limited information is available to predict failure after specific therapy, possibly guiding high-risk patients into tailored follow-up protocols (Pound *et al*, 1999; Kattan *et al*, 2001). In the presented study, we ought to analyse a soluble fragment of e-cadherin for its potential use as a serum biomarker in prostate cancer disease.

E-cadherin is a cell adhesion molecule playing a major role in maintaining proper epithelial cell-to-cell structure (Takeichi, 1990). This member of the cadherins, also called uvomorulin, is a 120 kDa transmembrane gylcoprotein. A very early report about the association between altered cell adhesion and tumour progression was published by a pathologist almost 60 years ago (Coman, 1944). After 40 years, reports of e-cadherin cleavage emerged when Wheelock and Damsky presented the discovery of a 80 kDa species of e-cadherin based on cell culture experiments (Wheelock *et al*, 1987). This proteolytic fragment, detectable in the media of tumour cells, was derived from the amino-terminal end of e-cadherin and could be reproducibly generated by a series of stress stimuli such as starving or high calcium loading. This fragment was thought to act proinvasive due to its ability to disrupt the cell–cell adhesion of mammary epithelium (Wheelock *et al*, 1987; Noe *et al*, 2001). In several previous studies including our own, decreased e-cadherin expression either alone or in combination with other biomarkers has been described as being associated with prostate cancer progression (Umbas *et al*, 1992; Takeichi, 1993; Rubin *et al*, 2001; Rhodes *et al*, 2003). We also reported that increased cleavage of the full-length 120 kDa e-cadherin, leading to 97 and 100 kDa fragments, was observed in neoplastic prostate tissue (Rashid *et al*, 2001; Rios-Doria *et al*, 2002). In a recent study, a considerable accumulation of the 80 kDa fragment in the extracellular compartment of metastatic prostate tissue has been observed (Kuefer *et al*, 2003). Several potential mechanisms have been discussed in the literature for being responsible for the shedding of e-cadherin (Takeichi, 1988; Katayama *et al*, 1994b; Banks *et al*, 1995; Noe *et al*, 2001; Ryniers *et al*, 2002). Still, the underlying pathomechanics need further investigation as the cleaved product of e-cadherin represents most likely only a bystander effect. Once these mechanisms, which lead to the cleaved product, are identified, specific therapy might be initiated with the potential use of the e-cadherin fragment as a marker for successful therapy.

Several studies, all using HECD-1 as the primary antibody for detection of the 80 kDa fragment, have reported the presence of the 80 kDA fragment in the serum of patients suffering from intestinal or bladder cancer.

The amount of detectable soluble 80 kDa e-cadherin was significantly higher in patients with *gastric cancer* compared to healthy individuals (Katayama *et al*, 1994a; Gofuku *et al*, 1998; Chan *et al*, 2001). Indeed, the 80 kDa serum levels were more sensitive for malignancies than well-known tumour markers, the carcinoembryonic antigen and CA19-9, a ligand of E-selectin (Gofuku *et al*, 1998). In a very recent study, the serum concentrations were an independent predictive factor for long-term survival in patients with gastric cancer (Chan *et al*, 2003). This observation and the fact that the expression levels did not correlate to clinical parameters such as T-stage is in accordance with the results described in this study. In contrast to our findings, for gastric cancer, a quick decline within already 7 days after surgery was observed (Gofuku *et al*, 1998). It was stated that a high concentration in gastric cancer patients predicted palliative treatment as there was a strong association to metastatic disease (Chan *et al*, 2001). Although colon cancer is an intestinal neoplasia as well, Velikova *et al* (1998) could not confirm these findings for colon cancer.

The soluble 80 kDa fragment was not only measured in the serum but also in the urine of patients. In one study, it was assumed that the 80 kDa fragment is excreted into the urine as increased levels could be measured among patients with different primaries (Katayama *et al*, 1994a). In *bladder cancer*, Banks *et al* described the existence of soluble forms of e-cadherin in the urine. An enhanced shedding and thus increased fraction of e-cadherin fragments was discussed as a loss of e-cadherin function in malignant changes of the bladder (Banks *et al*, 1995). This observation was put in perspective to the fact that decreased e-cadherin expression has been shown to be associated with poor survival in bladder cancer patients (Bringuier *et al*, 1993; Syrigos *et al*, 1995). In a more recent study, the soluble e-cadherin was included in a protein/creatinine index. This index showed a significant difference between invasive and noninvasive bladder cancers. Still, it was concluded that this protein alone most likely will not be useful for bladder cancer screening (Protheroe *et al*, 1999). Another study investigated the serum expression at the time of presentation and found significantly higher expression levels in patients with high-grade and muscle invasive bladder tumours. The most interesting finding for bladder cancer, which is in accordance with the presented results for prostate cancer, was the fact that patients at risk of early relapse of superficial bladder tumours had significantly elevated serum levels of soluble e-cadherin at presentation (Griffiths *et al*, 1996).

In this study, we demonstrated in a broad spectrum of prostate cancer disease stages and in comparison to healthy individuals and benign hyperplasia of the prostate, a very strong association of the 80 kDa fragment in the serum with neoplastic changes of the gland. The highest expression levels are seen in patients with advanced hormone refractory metastatic prostate cancer. The 80 kDa fragment in this setting was independent of any clinical parameters, including those related to tumour burden, in the cohort of clinically localised PCA cases. This observation is surprising, but may suggest that the 80 kDa e-cadherin fragment reflects biological character of the underlying cancer. The tumour biology obviously could be directly related to metastatic potential and thus to late recurrence after specific therapy. This hypothesis may explain the fact that in this setting patients with Loc PCA and a high 80 kDa serum level at the time of diagnosis have a considerable high risk of biochemical failure after surgery starting at a follow-up time of 3 years. To gain further insight into the underlying pathomechanics and to understand better why there is no association with tumour burden, a matched pairs analysis of serum levels of the fragment and e-cadherin expression in tissue samples might be helpful (Griffiths *et al*, 1996). Although further work is needed, these observations, in conjunction with the high serum expression levels in patients with far advanced lethal PCA, make the authors believe that the 80 kDa expression level has the potential to serve as biomarker with predictive value. As stated above, a real value for the urologist most likely could be its application in conjunction with other well-described parameters after cancer-specific therapy. It also would be very important to test lymph node-positive prostate cancer patients for their 80 kDa serum levels as these patients are more likely to fail during follow-up and therefore would benefit most from a biomarker with predictive value. Although this study refers to a decent sample size, a limitation is the low number of events (*n*=21), which may explain why PSA was not a predictor of failure in the cohort of localised PCA cases in this setting and which results in a limited degree of freedom in regression models. Consecutively, this serum marker needs to be approved in a larger and ideally prospective setting. Based on valid clinical interpretation, it may then help to tailor the follow-up regimen in patients being at high risk of failure. In upcoming studies, the end point preferably should not be biochemical failure, as an association to clinical recurrence or disease-specific death is most important and needs to be demonstrated. As there was no change in the serum levels between time of diagnosis and serum levels after surgery, the 80 kDa fragment may also serve as a marker for patients undergoing other therapeutic means for localised PCA.

The management of a patient with prostate cancer requires that prognostic information is available for defining an optimised treatment plan. The presented results are promising that the 80 kDa fragment of e-cadherin can be approved as a serum biomarker for prostate cancer, being detectable at early stages of disease before specific therapy is initiated and potentially providing additional prognostic information besides clinical and pathological data.

## Figures and Tables

**Table 1:** Demographics of all patients who were evaluated for the expression of the 80 kDa fragment of e-cadherin in the serum

	**Cases**	**Mean**	**Median**	**Range**
*Healthy volunteers (n*=*13)*				
Age (years)	13	26.3	27.0	22.0–32.0
				
*BPH (n*=*29)*				
Age (years)		63.6	63.0	60.0–68.0
Negative biopsies	29			
				
*Loc PCA*				
Nonfailures/failures (*n*=109/*n*=21)				
Age (years)	109/21	59.7/62.0	60.0/61.0	36.0–83.0/54.0–73.0
PSA (ng ml^−1^)		9.8/11.0	6.7/7.0	0.1–90.8/0.2–43.3
Gland weight (g)		61.7/50.4	53.0/42.0	28.2–131/26.3–94.0
Follow-up (days)		934.2/1659.6	867.0/1706.0	41–2226/171–2706
Tumour stage				
1	14/1			
2	74/2			
3	12/8			
4	8/7			
Gleason sum				
Unknown	3/2			
6	45/1			
7	56/11			
8	2/3			
9	3/4			
Multifocality				
0	26/4			
1	83/17			
Extraprostatic extension				
0	93/6			
1	16/15			
Surgical margin				
0	89/8			
1	20/13			
Seminal vesicle involvement				
0	101/14			
1	8/7			
				
*Met PCA (n*=*16)*				
Age at diagnosis (years)	16	65.2	68.0	57.0–79.0
Months to death after diagnosis		84.5	70.0	15.0–190.0
Gleason sum at diagnosis				
<6	3			
7–8	9			
9–10	4			

BPH=benign prostatic hyperplasia; Loc PCA=localised prostate cancer; Met PCA=metastatic prostate cancer; PSA=prostate-specific antigen.

**Table 2:** Clinical parameters and the concentration of the 80 kDa serum fragment are tested in an univariate and a multivariate regression model for association with outcome

**Variable**	***P*-value**	**Hazard ratio**	**95% CI**
*Univariate Cox's proportional-hazard model*			
Race	0.49	1.16	(0.76–1.79)
Age (years)	0.23	1.03	(0.78–2.14)
Preoperative PSA (ng ml^−1^)	0.83	1.004	(0.97–1.05)
Gland weight (g)	0.17	0.98	(0.95–1.0)
Gleason sum	<0.001	2.49	(1.62–3.84)
Multifocality	0.67	1.27	(0.43–3.78)
Extraprostatic extension	<0.001	10.94	(4.22–28.37)
Seminal vesicle involvement	0.002	4.34	(1.74–10.81)
Surgical margin	0.001	4.66	(1.91–11.36)
Tumour stage	<0.001	2.67	(1.69–4.22)
80 kDa e-cadherin fragment	0.79	1.12	(0.45–2.65)
			
*Multivariate (backward stepwise; Wald)*			
Gleason sum	0.007	2.16	(1.24–4.25)
Extraprostatic extension	0.007	5.34	(1.60–17.84)

95% CI=95% confidence interval; PSA=prostate-specific antigen.
